# Developing a comprehensive definition of sustainability

**DOI:** 10.1186/s13012-017-0637-1

**Published:** 2017-09-02

**Authors:** Julia E. Moore, Alekhya Mascarenhas, Julie Bain, Sharon E. Straus

**Affiliations:** 1 Li Ka Shing Knowledge Institute, St. Michael’s Hospital, 30 Bond Street, Toronto, ON M5B 1W8 Canada; 2 Department of Medicine, Faculty of Medicine, University of Toronto, 1 King’s College Circle, Medical Sciences Building, Toronto, ON M5S 1A8 Canada

**Keywords:** Sustainability, Sustainment, Routinization, Institutionalization, Maintenance, Evidence-based programs, Implementation strategies, Evolution

## Abstract

**Background:**

Understanding sustainability is one of the significant implementation science challenges. One of the big challenges in researching sustainability is the lack of consistent definitions in the literature. Most implementation studies do not present a definition of sustainability, even when assessing sustainability. The aim of the current study was to systematically develop a comprehensive definition of sustainability based on definitions already used in the literature.

**Methods:**

We searched for knowledge syntheses of sustainability and abstracted sustainability definitions from the articles identified through any relevant systematic and scoping reviews. The constructs in the abstracted sustainability definitions were mapped to an existing definition. The comprehensive definition of sustainability was revised to include emerging constructs.

**Results:**

We identified four knowledge syntheses of sustainability, which identified 209 original articles. Of the 209 articles, 24 (11.5%) included a definition of sustainability. These definitions were mapped to three constructs from an existing definition, and nine new constructs emerged. We reviewed all constructs and created a revised definition: (1) *after a defined period of time,* (2) *a program, clinical intervention, and/or implementation strategies continue to be delivered and/or* (3) *individual behavior change (i.e., clinician, patient) is maintained;* (4) *the program and individual behavior change may evolve or adapt while* (5) *continuing to produce benefits for individuals/systems.* All 24 definitions were remapped to the comprehensive definition (percent agreement among three coders was 94%). Of the 24 definitions, 17 described the continued delivery of a program (70.8%), 17 mentioned continued outcomes (70.8%), 13 mentioned time (54.2%), 8 addressed the individual maintenance of a behavior change (33.3%), and 6 described the evolution or adaptation (25.0%).

**Conclusions:**

We drew from over 200 studies to identify 24 existing definitions of sustainability. Based on these definitions, we identified five key sustainability constructs, which can be used as the basis for future research on sustainability. Our next step is to identify sustainability frameworks and develop a meta-framework using a concept mapping approach to consolidate the factors and considerations across sustainability frameworks.

**Electronic supplementary material:**

The online version of this article (doi:10.1186/s13012-017-0637-1) contains supplementary material, which is available to authorized users.

## Background

Understanding how to address issues of sustainability is “one of the most significant translational research problems of our time” [1]. To achieve lasting effects on health, it is essential to sustain the implementation of evidence and the ensuing outcomes. In their 2004 systematic review on implementation, Greenhalgh and colleagues noted the “near absence of studies focusing primarily on the sustainability of complex service innovations” [2]. Since then, the literature continues to grow, providing evidence-based guidelines [3–5], registries of evidence-based programs [6, 7], systematic reviews on the effectiveness of implementation strategies, and organized lists of implementation strategies [8–10]. However, much of the literature on sustainability remains theoretical, with little guidance on how to sustain program (or the clinical intervention) delivery, implementation strategies, and outcomes [11, 12].

Two of the foundational challenges of sustainability are the lack of a standard definition for the term and the variety of synonyms that are used in the literature [1]. Without a standard, widely accepted definition, it is unclear how researchers operationalize and measure sustainability. Indeed, in their development of a research agenda in sustainability, Proctor and colleagues prioritized improving clarity in sustainability terminology and concepts [1]. The use of different terms leads to challenges in finding the literature on sustainability, which in turn hamper the ability of researchers to grow this science and avoid duplication of effort. Commonly used alternative terms for sustainability include maintenance, continuation, institutionalization, routinization, and durability [11, 13]. These definitional challenges have likely arisen because multiple disciplines (e.g., medicine, health systems, child welfare, prevention science, education, justice, and juvenile justice) [14] are addressing similar problems.

Most implementation studies do not include an explicit definition of sustainability [11]. For example, in an early systematic review of sustainability, published in 2005, Scheirer noted that few studies provided a definition [13]. In 2016, Tricco and colleagues found that 8 (13%) of the 62 studies in their scoping review of interventions in healthcare decision-making related to chronic disease management included a definition of sustainability [12]. In their systematic review on the sustainability of clinical practice guidelines, Ament and colleagues noted that only 2 (11%) of the 18 studies provided a reference for their respective definitions of sustainability [15]. One explanation for the lack of referenced definitions could be that selecting a definition can be challenging, given that sustainability can refer to the sustained delivery of a clinical intervention or individual changes in behavior and can occur at multiple levels (e.g., patient, provider, organization, community, system) and given that commonly used definitions describe different constructs (see Table 1).

**Table 1:** Sustainability definitions

“Sustainability of organizational innovations can be thought of as the point at which new ways of working become the norm and the underlying systems and ways of working become transformed in support” [2].
“The simplest definition of sustainability is the ‘capability of being maintained at a certain rate or level’” [20].
“We use the term sustainment to denote the continued use of an innovation in practice” (Aarons et al. [33]).
“(1) [W]hether, and to what extent, the core elements (the elements mostly closely associated with desired health benefits) are maintained; (2) the extent to which desired health benefits are maintained and improved upon over time after initial funding or supports have been withdrawn; (3) the extent, nature, and impact of modifications to the core and adaptable/peripheral elements of the program or innovation; (4) continued capacity to function at the required level to maintain the desired benefits” [11].

Building on existing sustainability research and the recommendations from Proctor and colleagues, our goal for the current study was to systematically develop a comprehensive definition of sustainability based on definitions already used in the literature [1].

## Methods

### Identification of existing definitions

We conducted a search for knowledge syntheses of sustainability in healthcare interventions using the validated search filter for reviews in PubMed Clinical Queries (July 2016). Search terms were sustainability, sustainment, durability, fidelity, institutionalization, routinization, longitudinal, and long-term. We retrieved the included studies from each systematic or scoping review and identified those studies that included a definition of sustainability. We exclusively studied definitions of the sustained implementation of evidence in the same setting, not on the scale-up or spread of evidence implementation to different settings. The inclusion criteria and search strategy for included studies in each knowledge syntheses is provided in Additional file 1.

### Data abstraction and analysis

**Fig. 1:**
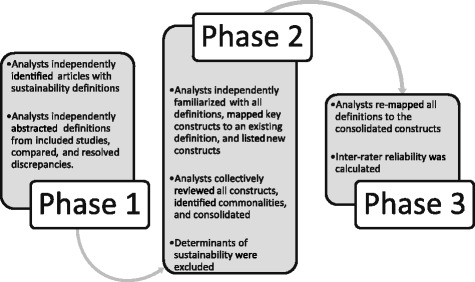
Abbreviated description of methods

We abstracted and mapped data from the articles within the knowledge syntheses across three phases. Figure 1 displays an abbreviated description of the steps in each phase.

### Phase 1: abstracting sustainability definitions from individual articles

Two analysts (AM, JB) identified articles that contained a definition of sustainability from the list of included studies in each knowledge synthesis. The same two analysts (AM, JB) then independently reviewed each eligible study to identify those that included a definition of sustainability. Two analysts then independently abstracted definitions from the included studies using a standard abstraction template. The list of included articles and sustainability definitions were then compared and discussed to resolve any discrepancies. In this phase, a discrepancy referred to (a) an instance where a study was included by one analyst and not the other or (b) an instance where one analyst extracted more or less textual detail than the other. The analysts resolved discrepancies by discussing whether the included studies and/or the included definitional content described the sustained implementation of evidence in a system, organization or community. Any studies or definitions pertaining only to adoption, implementation, spread of implementation, or scale-up of implementation in new settings were excluded.

### Phase 2: familiarizing with definitions and consolidating key constructs

Three analysts (JEM, AM, JB) conducted a familiarization activity [16–18]. In this activity, the analysts immersed themselves in the definitional content by reading and re-reading the list of phase 1 definitions and mapping new and recurrent constructs. Because a comprehensive definition of sustainability should build on existing definitions, the familiarization activity started with mapping content from phase 1 definitions to the definition of sustainability presented by Scheirer [13], which is based on the framework developed by Shediac-Rizkallah and Bone [19]. According to this definition, sustainability incorporates three key constructs: (1) continuation of health benefits for individuals after initial program funding ends, (2) continuation of program activities within one or more organizations, and (3) building of a community’s capacity to develop and deliver programs. The three analysts independently mapped the definitions to these three constructs (using a standard mapping template) and used open-text categories to map new and emergent constructs. Next, the three analysts collectively completed a side-by-side comparison of both (1) the three lists of definitions mapped to the Scheirer constructs and (2) the list of new constructs from each analyst. Because phase 2 was a familiarization activity, we did not calculate percent inter-rater agreement at this stage. The analysts identified commonalities across the constructs from the Scheirer definition and the list of new constructs and excluded constructs that did not define sustainability but rather described factors or determinants that affect sustainability. We used the following criteria to distinguish between defining constructs and determining constructs:

Constructs that *defined sustainability* included information about the characteristics of sustainability. For example, if the construct was measured it may provide proof that sustainability was happening in a given setting. These constructs were included in our study.

Constructs that were *determinants of sustainability* included information about potential influencers of sustainability. For example, the construct may promote or hinder sustainability, but its measurement may not provide proof that sustainability was occurring. These constructs were excluded from our study.

The final result of this phase was a consolidated list of new and emerging constructs. This list of constructs made up our comprehensive definition of sustainability; this definition was reviewed and approved by all authors.

### Phase 3: mapping definitions to the consolidated list of constructs

The phase 2 mapping template was revised to include the constructs in the comprehensive definition. The three analysts independently re-mapped all content from definitions into these consolidated constructs; no additional constructs were identified at this stage. Assumptions made between phases 2 and 3 of mapping are presented in Table 2. To assess the degree to which the three analysts consistently agreed on the re-mapped constructs, the percent agreement was calculated. The three analysts then discussed the re-mapped definitions and resolved any discrepancies. For the definitions retrieved from the reviews, we created counts and percentages of the definitions that included each of the constructs in our final definition.

**Table 2:** Assumptions for abstraction of sustainability definitions (phase 3)

• Any reference to time (e.g., “over time” or “after initial funding”) was coded as time (key construct 1).
• The continuation of a program, clinical intervention, innovation, implementation strategy, initiative, policy, project activity, or program component was categorized as organizational or community-based, unless the definition specified that such elements occurred at an individual level (key construct 2).
• The terms “practice change,” “ways of working,” and “individual routinization” were considered to represent maintenance of a behavior change by an individual (key construct 3).
• The use of terms such as “adaptation,” “evolution,” “modification,” and “variation” was coded as evolving or adapting (key construct 4).
• A broad range of terms was used to describe outcomes, including “benefits,” “effects,” “outcomes,” “performance goals,” and “program results” (key construct 5).

## Results

We identified four published knowledge syntheses of sustainability [11, 12, 15, 20]. Inclusion criteria varied across the four reviews. Gruen and colleagues included both conceptual frameworks and empirical studies about program sustainability within healthcare organizations or in community settings [20]. Stirman and colleagues searched for peer-reviewed studies that addressed the sustainability of specific interventions or programs, were written in English, and were published or in press by July 2011; they included all studies in which the authors used one of a predetermined set of terms to describe sustainability or made an effort to determine the extent to which a program or intervention continued after an initial period of training, implementation, or study [11]. Ament and colleagues included studies of sustainability that had at least two measurements (other than self-reporting), obtained before and immediately after implementation, of professionals’ adherence to a clinical practice guideline [15]. Tricco and colleagues included studies with an experimental, quasi-experimental, or observational (with one or more comparator groups) design, conducted in any clinical setting and involving adults with a chronic disease (excluding mental illness) who received a knowledge translation intervention (targeting the patient, a healthcare provider, or the health system) that lasted more than 1 year after implementation or termination of study funding [12]. The four reviews encompassed a total of 240 original research publications (39 from Gruen and colleagues [20], 125 from Stirman and colleagues [11], 14 from Ament and colleagues [15], and 62 from Tricco and colleagues [12]); because of overlap among the reviews, there were 209 unique citations.

### Phase 1: abstracting sustainability definitions from individual articles

In phase 1, data abstraction, 24 (11.5%) of the 209 eligible articles provided a definition of sustainability.

### Phase 2: familiarizing with definitions and consolidating key constructs

In phase 2, the three reviewers identified a total of 94 constructs (32 by AM, 32 by JB, 30 by JEM), which were consolidated into nine constructs not included in the Scheirer definition. All three coders identified the following three constructs: institutionalization (i.e., establishment of a new practice, program, or clinical intervention that becomes the norm within an organization or other setting, such as a particular community; 29/94 [30.9%]), routinization (i.e., establishment of a new practice at an individual level; 20/94 [21.3%]), and adaptation or evolution (i.e., change in the nature of programs, implementation strategies, and individual behavior, in response to changes in the broader ecological context; 21/94 [22.3%]). The remaining six constructs were each identified by a single coder: time (9/94 [9.6%]), improvement trajectories (i.e., outcomes are not only maintained but steadily improve over time; 4/94 [4.3%]), benefits and partnerships with stakeholders (4/94 [4.3%]), maintenance of core elements of evidence-based programs (3/94 [3.2%]), implementation as sustainability (i.e., the implementers are both the means of achieving sustainability and part of the outcome; 2/94 [2.1%]), and interactions with the environment (i.e., achievement of sustainability not in isolation from the environment, but as a result of interactions between the program and the environment; 2/94 [2.1%]).

We reviewed all of these nine constructs and expanded our list of constructs for analysis. A key theme from the review of constructs was that some definitions considered the implementation of evidence-based programs (e.g., multidimensional treatment foster care [21]), whereas others focused on the implementation of a specific evidence-based practice (e.g., recommendations in a clinical practice guideline) that was not part of a program. Definitions referring to evidence-based programs primarily focused on continuing program delivery at the organizational level, using descriptions such as “the continued use of an intervention” [22], “program or policy becomes institutionalized or part of the routine organizational practices and policies” [23], “program components…are maintained” [13], or “core elements are maintained” [11]. Definitions that focused on evidence-based practices described maintenance of a behavior by individuals: “new ways of working…become the norm” [24], “new working methods…are maintained” [25], or “enduring part of the behavioral repertoire of an individual” [23]. Some of the discrepancies across definitions were related to different considerations of what was being implemented: individual behavior change or organizational programs. To address these two perspectives, our comprehensive definition includes constructs related to both maintenance of behavior change by individuals (e.g., program recipients, patients, caregivers, or clinicians) and continued delivery of a program at the organizational level (e.g., organizational or community-level implementation of programs).

Several definitions suggested that sustainability involves, in part, the evolution or adaptation of programs, implementation strategies, or practices over time. Examples included “a suggested ‘adaptation phase’ that integrates and institutionalizes interventions within local organizational and cultural contexts” [22], “the dynamism of continuing change” [26], and “creating an environment for innovations to adapt to the system, if necessary” [27]. Some of the definitions referred to “time,” either providing specific criteria (e.g., 2 years after end of funding) or using vague allusions (e.g., after initial funding ends). We believe that the timeline for sustainability depends on the individual practice or program and the outcomes of interest, so our definition does not specify a particular timeline; nonetheless, we believe it is essential for operational definitions of sustainability to specify a timeline. Therefore, our comprehensive definition of sutainability includes the following five constructs: (1) *after a defined period of time,* (2) *the program, clinical intervention, and/or implementation strategies continue to be delivered and/or* (3) *individual behavior change (i.e., clinician, patient) is maintained;* (4) *the program and individual behavior change may evolve or adapt while* (5) *continuing to produce benefits for individuals/systems.*

### Phase 3: mapping definitions to the consolidated list of constructs

In phase 3, we re-mapped the new sustainability constructs from the definitions (Table 3). Inter-rater reliability for the mapped constructs was 94%, indicating substantial agreement among the three reviewers [28]. Of the 24 definitions, 17 (71%) described the continued delivery of a program, 17 (71%) mentioned continued benefits or outcomes, 13 (54%) mentioned time, 8 (33%) addressed maintenance of behavior change by individuals, and 6 (25%) described evolution or adaptation of a behavior or practice. Additional file 2 provides quotes from the source definitions describing each of the five constructs to allow future researchers to use alternative wording for individual constructs in adapting our definition to their own projects.

**Table 3:** Summary of definition references abstracted to sustainability constructs

Definition references	Time	Continued delivery	Behavior change	Evolution/adaptation	Continued benefits
Aarons et al. (2011) [33]			X		
Blasinsky et al. [34]	X	X			
Bossert (1990) [35]	X	X			X
Buchanan et al. (2005) [25]	X		X		X
Chambers et al. (2013) [22]	X	X		X	X
Doyle et al. (2013) [36]			X		X
Evashwick and Ory (2003) [37]	X	X			
Fleiszer et al. (2015) [26]	X	X		X	X
Glasgow et al. (1999) [23]	X	X	X		X
Goodman et al. (1993) [38]		X			
Greenhalgh et al. (2004) [2]			X		
Gruen et al. (2008) [20]		X			X
Johnson et al. (2004) [27]		X		X	X
Mancini and Marek (2004) [39]		X			X
National Health Service (2007) [24]	X		X	X	X
Olsen (1998) [40]					X
Paine-Andrews et al. (2000) [30]	X	X	X		
Pluye et al. (2004) [41]		X		X	
Scheirer (2005) [13]	X	X			X
Schell et al. (2013) [42]	X	X			X
Shediac-Rizkallah and Bone (1998) [19]		X			X
Stetler et al. (2007) [43]	X		X		X
Stirman et al. (2012) [11]	X	X		X	X
Swerissen and Crisp (2004) [44]		X			X

## Discussion

We identified five key constructs that describe individual and organizational sustainability. Our definition of sustainability was crafted to encompass these five key constructs: (1) *after a defined period of time,* (2) *the program, clinical intervention, and/or implementation strategies continue to be delivered and/or* (3) *individual behavior change (*i.e.*, clinician, patient) is maintained; (4) the program and individual behavior change may evolve or adapt while (5) continuing to produce benefits for individuals/systems.*

The five constructs are distinct yet inter-related. For example, continued delivery of a clinical intervention or program (i.e., institutionalization) refers to whether an organization or community is continuing to provide a program (e.g., delivering multidimensional treatment foster care) or continuing to use the strategies necessary to support behavior change (e.g., education, audit, and feedback). Maintenance of behavior change at the individual level pertains to whether the implementer is following the recommendations of the evidence-based program, guideline, or practice (e.g., how the implementer is interacting with patients, clients, or community members). Both institutionalization and maintenance may evolve over time. The advantage of implementing evidence-based programs, rather than newly developed and untested programs, is that if such programs are delivered with high implementation quality, they can be expected to produce anticipated outcomes [29]. However, initial program outcomes will not necessarily continue following the implementation phase; therefore, maintaining outcomes is considered a separate construct, one that should be measured separately from program delivery and maintenance of behavior change. Institutionalization, maintenance, and benefits can each be defined and measured over a period of time.

Continued delivery of a program or a set of implementation strategies (i.e., institutionalization) and maintenance of benefits were the most commonly included constructs. The prominence of these two constructs (cited together in 12 [50%] of the 24 definitions) is not surprising, given that the majority of individual studies focused on the implementation and sustainability of evidence-based programs, rather than individual-level practice changes. Only 2 (8%) of the definitions incorporated both maintenance of a behavior by individuals and continued delivery of the program [21, 30].

Just over half of the definitions (13/24 [54%]) mentioned the word “time.” However, all but three of these time-related references were undefined (e.g., “over time” or “after funding ended”); continued program delivery, maintenance of behavior change, and outcomes can all be measured over time. Depending on the nature of the intervention and its outcomes, the appropriate time to start measuring sustainability will vary.

Evolution and/or adaptation constituted the least commonly described construct, mentioned in 25% of the definitions. Adaptation can refer to either changes in the program or implementation strategies or changes in an individual’s maintenance of a behavior. Given that sustainability frameworks, such as the dynamic sustainability framework [22], strongly emphasize the evolutionary nature of evidence, programs, and practices, it seemed imperative to capture this concept in the definition.

Some definitions included the related assumptions that researchers were measuring the continuation of a program and that initial implementation would be affected by context but the program itself (or maintenance of the behavior) would not change because of context [23]. In these cases, contextual factors are conditions that may increase or decrease the likelihood of sustainability [11]. Some definitions described the program’s level of stability [23]. Other researchers approached sustainability from more of an ecological perspective, whereby the program and the environment were perceived as being interconnected [13, 22]. These various approaches may yield different research study questions: (1) factors or determinants affecting sustainability or (2) ways in which the environment and program adapt and evolve together. Additionally, each of these approaches has significant implications for how people plan for sustainability.

We flagged several constructs that are associated with sustainability, for example, factors that affect the evolution of a program or practice over time. These were similar to the influences on sustainability identified by Stirman and colleagues in their review, which included the following broad categories: innovation characteristics, context, capacity, processes, and interactions [11]. We did not include these predictive constructs in our definition of sustainability, although they are integral to an understanding of evolution and dynamism and should be considered when planning for and measuring sustainability.

Fleiszer and colleagues completed a concept analysis on the sustainability of healthcare innovations where they described three characteristics of sustainability: continued benefits, routinization/institutionalization, and development [26]. Authors described their conceptualization of sustainability as needing further development prior to measurement or testing. Our comprehensive definition builds on this work by distinguishing between routinization (i.e., individual level change) and institutionalization (organization/system level change) and by including the construct of time.

The lack of a comprehensive definition of sustainability has been a foundational challenge to moving the field forward because a definition may help implementers think about *what* it is they hope to sustain [12]. In a recent systematic review on the sustainability of health interventions in sub-Saharan Africa [31], only half of the studies (*n* = 21, 51.2%) clarified what it is they are sustaining using different definitions from the literature. The majority of all studies (*n* = 19, 46.3%) reported outcomes on the continued delivery of a program (46.3%). Similar results were found in a scoping review on the sustainability of chronic disease health programs [32] the majority of studies (*n* = 37, 88.1%) included sustainability indicators related to the maintenance of program activities. Furthermore, the system or organization was the unit of analysis for the majority of studies, with only 23.8% (*n* = 10) of studies measuring individual-level sustainability outcomes [32]. These results suggest a more narrow understanding of sustainability than what we outlined in our definition in the current study. Our comprehensive definition of sustainability prompts implementers to think about what it is they hope to sustain at the individual level, the organization/system level, and the level of intervention outcomes.

A few limitations of this study should be noted. First, we did not conduct a systematic search for articles about sustainability; rather, we identified primary articles only from the existing knowledge syntheses. Inclusion criteria varied within each knowledge syntheses, and therefore, we might have missed some articles that included definitions of sustainability. In particular, newer articles (published in 2015 and 2016) are not likely to have been identified by our method, and the search was limited to articles published in English. Second, this search of knowledge syntheses was only performed in one healthcare database. There may be knowledge syntheses on sustainability in literature outside of healthcare (e.g., social services research) that could have been excluded from our study, and there may be knowledge syntheses we missed from other healthcare databases. Third, given differing perspectives on sustainability (e.g., the ecological approach emphasizing evolution and the determinant approach focusing on stability), other researchers analyzing the same literature might have drawn different conclusions about the constructs to be included in the definition. Future research could build on our work by conducting a more comprehensive search for sustainability definitions and then testing the constructs from our work in that literature.

## Conclusions

Based on a reviewing article identified through the four knowledge syntheses on sustainability, we developed a comprehensive definition of sustainability that includes five constructs: (1) *after a defined period of time,* (2) *the program, clinical intervention, and/or implementation strategies continue to be delivered and/or* (3) *individual behavior change (i.e., clinician, patient) is maintained;* (4) *the program and individual behavior change may evolve or adapt while* (5) *continuing to produce benefits for individuals/systems.* The next step for the mapped definitions and sustainability frameworks will be to use a concept mapping approach to develop a meta-framework and to thereby consolidate factors and considerations across sustainability frameworks. A tool will then be developed to support researchers and implementers in operationalizing the meta-framework.

## Additional files

Additional file 1: Characteristics of included reviews. (DOCX 18 kb)

Additional file 2: Definitions abstracted to sustainability constructs. (DOCX 24.3 kb)
