# Supplementary material for: Developing a comprehensive definition of sustainability
Source: Implement Sci. 2017 Sep 2;12:110. doi: 10.1186/s13012-017-0637-1 (PMC5581411; doi:10.1186/s13012-017-0637-1)
Supplement: Supplementary file 1 — Characteristics of included reviews. (DOCX 18 kb) [file 13012_2017_637_MOESM1_ESM.docx]

**Additional file 1:** Characteristics of included reviews

**AMSTAR criteria**

|  | **Ament 2015** | **Gruen 2008** | **Stirman 2012** | **Tricco 2016*** |
| --- | --- | --- | --- | --- |
| An ‘a priori’ designed provided | no | no | no | yes |
| Duplicate study selection | yes | yes | no | yes |
| Comprehensive literature search performed | yes | yes | yes | yes |
| Status of publication used as an inclusion criterion | yes | Not clear | Not clear | yes |
| A list of included and excluded studies provided | yes | no | no | yes |
| Characteristics of included studies provided | yes | no | no | yes |
| Scientific quality of included studies assessed and documented | yes | no | no | n/a for scoping reviews |
| Scientific quality of the included studies used appropriately in formulating conclusions | yes | no | no | n/a for scoping reviews |
| Methods used to combine findings of studies is appropriate | yes | yes | yes | yes |
| Likelihood of publication bias is assessed | no | no | no | n/a for scoping reviews |
| Conflict of interest is stated | yes | yes | yes | yes |
| **AMSTAR RATING** | 9 | 4 | 3 | 8/8 |

**This is a scoping review, not a systematic review so not all AMSTAR elements are relevant*

**Search strategy and inclusion criteria**

| **Gruen, 2008** |
| --- |
| **Search Terms:** “programme sustainability”, “continuation”, “institutionalisation”, “resilience”, “durability”, “viability”, “stability”, “persistence”, and “main tenance”; We then restricted the search to citations included under health-care organisation and community-care MeSH headers, and not included under the agriculture MeSH headers. |
| **Databases searched:** Medline (1980 to June 18, 2008), EmBase (1950 to June 18, 2008), and the Cochrane library |
| **Inclusion Criteria:** Sustainability of health system programmes |
| **Exclusion Criteria:** Not provided |

| **Stirman, 2012** |
| --- |
| **Search Terms:** “sustainability,” “implementation,” “long-term implementation,” “routinization,” “discontinuation,” “de-adoption,” “durability,” “institutionalization,” “maintenance,” “capacity building,” and “knowledge utilization.” Truncated forms of these terms (e.g., “sustai*”, “routini*”, “institutionali*”) and alternative spellings were included in the search. |
| **Databases searched:** MEDLINE, ISI, PsycINFO, Academic Search Premier, Health Source, ERIC, and Google Scholar |
| **Inclusion Criteria:** Our inclusion criteria included peer-reviewed studies that addressed sustainability of specific interventions or programs, were written in English, and were published or in press by July 2011. Because sustainability has been defined in numerous ways, we included all studies in which the authors used one of the terms described above or in which an effort was made to determine the extent to which a program or intervention continued after an initial period of training, implementation, or study. Studies were coded only if they included a methodology or procedure designed to identify (1) the status of the program after the initial implementation effort or funding has ended (e.g., fidelity, percent implemented, presence or absence of key components, or discontinuation); (2) the program-, service-, or recipient-level outcomes measured after external support or funding was withdrawn; or (3) the influences on the persistence of the implementation, whether or not the primary focus of the article was sustainability. |
| **Exclusion Criteria:** Articles were excluded if they (1) reported only on initial implementation efforts, (2) were purely narrative accounts or papers on “lessons learned” that did not examine sustainability using qualitative or quantitative research methodologies, (3) reported only long-term follow-up of individuals after a clinical trial or intervention study, or (4) contained insufficient information to determine whether inclusion or exclusion criteria were met (e.g., ambiguity or failure to report the timeframe during which measures were collected). Studies were considered to focus on initial implementation efforts if the original training, supervision, monitoring, or funding support was ongoing throughout the time period of the research (unless monitoring was considered a central element of the program or conducted strictly to assess sustainability, with minimal or no feedback provided). |

| **Ament, 2015** |
| --- |
| **Search Terms:** Free-text terms and MeSH terms regarding sustainability, quality improvement, impact and guideline recommendations were used. |
| **Databases searched:** MEDLINE (OvidSP; 1946 to February 2014), CINAHL (EBSCO Host; 1982 to February 2014), EMBASE (OvidSP; February 2014), Cochrane Central Register of Controlled Trials (CENTRAL) and the Guidelines International Network (GIN) library for studies |
| **Inclusion Criteria:** Studies needed to be focused on sustainability and on clinical practice guidelines. Sustainability was described as “Sustainability of change exists when a newly implemented innovation continues to deliver the benefits achieved over a longer period of time, certainly does not  return to the usual processes and becomes ‘the way things are done around here’,11 even after the implementation project is no longer actively carried out, until a better innovation comes along”.12 Studies had to include at least two measurements: one before (PRE) or immediately after implementation (EARLY POST) and one measurement longer than 1 year after active implementation (LATE POST). All activities to facilitate the adherence to clinical practice guidelines were labeled as part of the implementation project. Studies needed to be focused on professionals’ adherence to a clinical practice guideline. Lastly, studies had to focus on medical care. Participants had to be healthcare professionals who deliver direct patient care. There were no restrictions on study design of the research articles. |
| **Exclusion Criteria:** Studies only using self-reported adherence were excluded to reduce the chance of social desirability bias and an overestimation of results. |

| **Tricco, 2016** |
| --- |
| **Search Terms:** durability, fidelity, sustainability, institutionalization, routinization, longitudinal and long-term |
| **Databases searched:** MEDLINE, Embase, Cochrane Central Register of Controlled Trials (CENTRAL), Cumulative Index to Nursing and Allied Health Literature (CINAHL) and the Campbell databases from inception until February 2013 |
| **Inclusion Criteria:** We included studies that targeted adults with chronic disease who received a KT intervention (which may have targeted the patient, their healthcare provider, or the health system). Studies including patients with chronic diseases but without specifying the conditions were included. All comparators were eligible for inclusion, such as other KT interventions or usual care. The study designs included were experimental (randomized controlled trials (RCTs), quasi-RCTs, non-RCTs), quasi-experimental (controlled before after studies, interrupted time series), and observational studies with a comparator group(s) (i.e. comparative cohort and case control studies). Studies lasting more than 1 year after implementation or the termination of the study funding across all clinical settings were included. |
| **Exclusion Criteria:** Studies on mental illness were excluded. Authors also excluded studies that were not on chronic disease, that had no relevant comparators, no KT intervention, and the time period was less than a year after implementation. |
