# Supplementary material for: Developing a comprehensive definition of sustainability
Source: Implement Sci. 2017 Sep 2;12:110. doi: 10.1186/s13012-017-0637-1 (PMC5581411; doi:10.1186/s13012-017-0637-1)
Supplement: Supplementary file 2 — Definitions abstracted to sustainability constructs. (DOCX 24.3 kb) [file 13012_2017_637_MOESM2_ESM.docx]

**Additional file 2: Definitions abstracted to sustainability constructs**

| **Reference** | **Synonym** | **Definition** | **After a period of time** | **Continued delivery of a program, clinical intervention and/or implementation strategies** | **Maintenance of behavior change by individuals** | **Evolution or adaptation** | **Continued benefits or outcomes** | **No. of constructs** |
| --- | --- | --- | --- | --- | --- | --- | --- | --- |
| **Aarons et al. (2011) [33]** | Sustainment | We use the term sustainment to denote the continued use of an innovation in practice. |  |  | X |  |  | 1 |
| **Blasinsky et al. [34]** | Sustainability | The continuation of all or part of the program after initial external funding ends. | X | X |  |  |  | 2 |
| **Bossert (1990) [35]** | Sustainability | Projects were considered to be sustained if the project activities and benefits continued at least 3 years after the life of the project. Benefits (project outcomes) are the intended or unintended positive impacts of the activities for the health of the national populations. | X | X |  |  | X | 3 |
| **Buchanan et al. (2005) [25]** | Sustainability | The sustainability of change can be broadly defined as the process through which new working methods, performance goals and improvement trajectories are maintained for a period appropriate to a given context. | X |  | X |  | X | 3 |
| **Chambers et al. (2013) [22]** | Sustainability | Sustainability is the extent to which an evidence-based intervention can deliver its intended benefits over an extended period of time after external support from the donor agency is terminated …. Sustainment is the continued use of an intervention within practice. Sustainability has evolved from being considered as the endgame of a translational research process to a suggested 'adaptation phase’ that integrates and institutionalizes interventions within local organizational and cultural contexts. | X | X |  | X | X | 4 |
| **Doyle et al. (2013) [36]** | Sustainability | Sustainability in this context means the continuation or the integration of new practice within an organization whereby it has become a routine part of care delivery and continues to deliver desired outcomes |  |  | X |  | X | 2 |
| **Evashwick and Ory (2003) [37]** | Sustainability | The endurance of the program since its inception. | X | X |  |  |  | 2 |
| **Fleiszer et al. (2015) [26]** | Sustainability | The term ‘sustainability’ is the broadest in meaning. It implies the stability of ingrained change and the dynamism of continuing change. Definitions of sustainability emphasize the following characteristics: innovation-related benefits; and/or the persistence of the innovation itself; and/or the development of the innovation or the context over time. The characteristics of innovation sustainability stem from the three main elements – namely, benefits, routinization/ institutionalization and development – identified in definitions. | X | X |  | X | X | 4 |
| **Glasgow et al. (1999) [23]** | Maintenance | Maintenance measures the extent to which innovations become a relatively stable, enduring part of the behavioral repertoire of an individual (or organization or community). OR The extent to which a program or policy becomes institutionalized or part of the routine organizational practices and policies. At the individual level, maintenance has been defined as the long-term effects of a program on outcomes after 6 or more months after the most recent intervention contact. | X | X | X |  | X | 4 |
| **Goodman et al. (1993) [38]** | Institutionalization | Institutionalization occurs when a program becomes an integral part of an organization, and the level of institutionalization (LoIn) instrument is a beginning effort to measure the extent of program integration into organizations. The instrument is based on theory that holds that organizations are composed of production, maintenance, supportive, and managerial subsystems. Institutionalization occurs when a program becomes imbedded into these subsystems. |  | X |  |  |  | 1 |
| **Greenhalgh et al. (2004) [2]** | Sustainability | Sustainability of organisational innovations can be thought of as the point at which new ways of working become the norm and the underlying systems and ways of working become transformed in support. Other definitions: Making an innovation routine until it reaches obsolescence OR implementing innovations in health service delivery and organisation and ensuring that they are sustained until they reach genuine obsolescence. |  |  | X |  |  | 1 |
| **Gruen et al. (2008) [20]** | Sustainability | The simplest definition of sustainability is the ‘capability of being maintained at a certain rate or level’. We propose that health-programme sustainability is the ultimate manifestation of a complex web of inter-relations between health concerns, stakeholders, resources, and actions analogous to an ecosystem. Sustainability is increased to the degree to which the components of the system are connected and aligned—an indication of system equilibrium. Sustainability or its absence can be shown by quantification of the continuation of health benefits, intervention, or capacity over time, but understanding the determinants of sustainability needs exploration of interactions between drivers and programme components in a particular context. Normative definitions of sustainability: maintenance of health benefits, continuation of health programmes, institutionalisation of programmes within organisational systems, community capacity, and multi-dimensional. |  | X |  |  | X | 2 |
| **Johnson et al. (2004) [27]** | Sustainability | Sustainability is the continued ability of an innovation (infrastructure or program) to meet the needs of its stakeholders central to the sustainability process. We define sustainability as the process of ensuring an adaptive prevention system and a sustainable innovation that can be integrated into ongoing operations to benefit diverse stakeholders. First, we view sustainability as a chance process with specific sustainability action steps to strengthen system infrastructure and innovation attributes that are necessary to sustain a particular innovation. Second, ensuring an adaptive prevention system is a part of the sustainability process. The system must be receptive to change, thus creating an environment for innovations to adapt to the system, if necessary, to which they are introduced. Third, 'what is to be sustained' is innovation (i.e., that which is new to the prevention system). Fourth, a sustainable innovation (i.e., program or infrastructure) is fully integrated into normal operations in that it has passed through the essential cycles and passages. Fifth, a sustainable innovation should be proven to be of benefit to the diverse stakeholders prior to the adoption and after implementation in a target prevention system. |  | X |  | X | X | 3 |
| **Mancini and Marek (2004) [39]** | Sustainability | Sustainability is the capacity of programs to continuously respond to community issues. A sustained program maintains a focus consonant with its original goals and objectives, including the individuals, families, and communities it was originally intended. There are three element dimensions to the conceptual framework logic model: elements associated with sustainability, middle-range program results, and an ultimate result of the program being sustained. We assume that sustainability elements lead to desired middle-range program results and that these desired results increase the chances of a program being sustained. We believe the conceptualization of sustainability is more complex than simply the question of whether a program continues to provide services. Rather than being seen as a dichotomy, sustainability might be more accurately measured via levels of program activity or via an assessment of the degree that current programs goals are consonant with original program goals and the degree to which program benefits are available to communities |  | X |  |  | X | 2 |
| **National Health Service (2007) [24]** | Sustainability | Sustainability is when new ways of working and improved outcomes become the norm. Not only have the process and outcome changed, but the thinking and attitudes behind them are fundamentally altered and the systems surrounding them are transformed in support. In other words it has become an integrated or mainstream way of working rather than something ‘added on’. As a result, when you look at the process or outcome one year from now or longer, you can see that at a minimum it has not reverted to the old way or old level of performance. Further, it has been able to withstand challenge and variation; it has evolved alongside other changes in the context, and perhaps has actually continued to improve over time. | X |  | X | X | X | 4 |
| **Olsen (1998) [40]** | Sustainability | A health service is considered sustainable when operated by an organizational system with the long-term ability to mobilize and allocate sufficient and appropriate resources (manpower, technology, information, and finance) for activities that meet individual or public health needs. The system is sustainable when it has the capacity to initiate desired changes or adapt to changes in demand or in environmental conditions, while ensuring resources and desired output. The framework includes three clusters: (1) contextual factors which outline the task and general environment for the services, (2) an activity profile, which describes the services delivered and activities carried out to deliver them; and (3) organizational capacity, which shows the carrying ability (capability) of the organization in broad terms. |  |  |  |  | X | 1 |
| **Paine-Andrews et al. (2000) [30]** | Sustainability | Sustainability was examined in two ways: (a) the extent to which community changes (i.e., new or modified programs, policies, or practices consistent with the mission of the initiatives) facilitated by the community health initiatives remained in place after grant termination and (b) the extent to which the initiatives themselves remained in place after grant termination. Community changes were defined as ongoing if they were reported to be taking place on a regular basis with no apparent end in sight. The initiative was determined to be in place if at least one staff person had responsibility for carrying out the mission or the initiative or if another group within the community adopted the initiative. | X | X | X |  |  | 3 |
| **Pluye et al. (2004) [41]** | Sustainability | The literature suggests that routinization is the primary or fundamental process in the sustainability of health promotion program. Sustainability is achieved through routinization, where the program is sustained, activities have resulted from it, and they have been routinized and their maintenance seems assured in the long term. There are four characteristics of organizational routines: (1) memory - routines become memorized, (2) adaptation - activities are adapted in accordance with their context, (3) values - routinized activities in organizations reflect collective values and beliefs, and (4) rules - routinized activities conform to rules governing decision-making and action. |  | X |  | X |  | 2 |
| **Scheirer (2005) [13]** | Sustainability | The program components developed and implemented in earlier stages are maintained after the initial funding or other impetus is removed. Three definitions of sustainability were examined: continued program activities, continued measured benefits or outcomes for new clients, and maintained community capacity. | X | X |  |  | X | 3 |
| **Schell et al. (2013) [42]** | Sustainability | Over time, a program ideally can sustain various elements, including its activities, community-level partnerships, organizational practices, benefits to its clients, and the salience of the program's core issue. We define sustainability capacity as the existence of structures and processes that allow a program to leverage resources to effectively implement and maintain evidence-based policies and activities. This definition is deliberately broad, and moves beyond the characteristics of a program itself that might support its sustainability to include organizational and systems characteristics. | X | X |  |  | X | 3 |
| **Shediac-Rizkallah and Bone (1998) [19]** | Sustainability | Sustainability thus appears to be a multi-dimensional concept of the continuation process and the term encompasses a diversity of forms that this process may take. … Important categories of sustainability indicators include: (1) maintenance of health benefits achieved through an initial program, (2) level of institutionalization of a program within an organization and (3) measures of capacity building in the recipient community. |  | X |  |  | X | 2 |
| **Stetler et al. (2007) [43]** | Sustainability | Changes (practice and outcomes) based on evidence that continue over time as related to specific projects. | X |  | X |  | X | 3 |
| **Stirman et al. (2012) [11]** | Sustainability | The continuation of an innovation within an organization or community. … A program or intervention's *impact* may be considered sustained if desired health benefits remain at or above the level achieved during implementation and this increase can be attributed to the program. A program or intervention may be considered sustained at a given point in time if, after the initial implementation support has been withdrawn, core elements are maintained (e.g., remain recognizable) or delivered at a sufficient level of fidelity or intensity to yield desired health outcomes and adequate capacity for continuation of these elements is maintained. Author suggests several factors to consider when choosing a definition of sustainability: (1) whether, and to what extent, the core elements (the elements mostly closely associated with desired health benefits), are maintained; (2) the extent to which desired health benefits are maintained and improved upon over time after initial funding or supports have been withdrawn; (3) the extent, nature, and impact of modifications to the core and adaptable/peripheral elements of the program or innovation; (4) continued capacity to function at the required level to maintain the desired benefits | X | X |  | X | X | 4 |
| **Swerissen and Crisp (2004) [44]** | Sustainability | For health promotion, sustainability may refer to intervention effects or the means by which these are produced—the programmes and agencies that implement interventions. The aim of health promotion is to produce intervention effects that may be sustained. In developing a policy on sustainability for health promotion, it is necessary to be able to differentiate between (i) levels of social organization which are the focus of change, (ii) the programmes and agencies which are the means to achieve change, (iii) the outcomes or effects that are achieved. |  | X |  |  | X | 2 |
| **No. (%) of definitions** |  |  | 13 (54%) | 17 (71%) | 8 (33%) | 6 (25%) | 17(71%) |  |
